# Dermoscopic Features of Facial Pigmented Skin Lesions

**DOI:** 10.1155/2013/546813

**Published:** 2013-02-03

**Authors:** Yana Goncharova, Enas A. S. Attia, Khawla Souid, Inna V. Vasilenko

**Affiliations:** ^1^Department of Dermatology, Venereology and Cosmetology, Donetsk Medical National University, Ukraine; ^2^Department of Dermatology, Queen Medical, Villaggio, P.O. Box 1418, Doha, Qatar; ^3^Department of Dermatology, Venereology and Andrology, Faculty of Medicine, Ain Shams University, P.O. Box 11381, Cairo, Egypt; ^4^Department of Pathology, Donetsk Medical National University, Ukraine

## Abstract

Four types of facial pigmented skin lesions (FPSLs) constitute diagnostic challenge to dermatologists; early seborrheic keratosis (SK), pigmented actinic keratosis (AK), lentigo maligna (LM), and solar lentigo (SL). A retrospective analysis of dermoscopic images of histopathologically diagnosed clinically-challenging 64 flat FPSLs was conducted to establish the dermoscopic findings corresponding to each of SK, pigmented AK, LM, and SL. Four main dermoscopic features were evaluated: sharp demarcation, pigment pattern, follicular/epidermal pattern, and vascular pattern. In SK, the most specific dermoscopic features are follicular/epidermal pattern (cerebriform pattern; 100% of lesions, milia-like cysts; 50%, and comedo-like openings; 37.50%), and sharp demarcation (54.17%). AK and LM showed a composite characteristic pattern named “strawberry pattern” in 41.18% and 25% of lesions respectively, characterized by a background erythema and red pseudo-network, associated with prominent follicular openings surrounded by a white halo. However, in LM “strawberry pattern” is widely covered by psewdonetwork (87.5%), homogenous structureless pigmentation (75%) and other vascular patterns. In SL, structureless homogenous pigmentation was recognized in all lesions (100%). From the above mentioned data, we developed an algorithm to guide in dermoscopic features of FPSLs.

## 1. Introduction

Until now, almost only melanocytic lesions selected on the basis of their clinical atypia or which appear equivocal on naked eye examination have been shown to benefit from the use of dermoscopy. In our experience, dermoscopic evaluation of pigmented lesions located on the face may require a different approach, as many of them are nonmelanocytic in nature. Moreover, at this site, dermoscopy reveals specific criteria according to the particular histological architecture shown by sun-damaged skin [1]. For example, under dermoscopy, the presence of a pseudonetwork is characteristic of pigmented nonmelanocyte lesions on the face. This feature may not be related to the rete ridges of the epidermis—which are absent or blunted due to the anatomy of the skin in this area and to photoaging—but is due to the interruption of the homogeneous pigmentation by the openings of hair follicle ostia and adnexal structures [2]. Hence, four types of facial pigmented skin lesions (FPSLs) constitute diagnostic challenge to dermatologists, namely, early seborrheic keratosis (SK), pigmented actinic keratosis (AK), lentigo maligna (LM), and solar lentigo (SL). We aimed to analyze the dermoscopic patterns of histopathologically diagnosed FPSLs to determine the distinguishing dermoscopic features of each of the aforementioned lesions to help reaching proper decisions regarding management of such an FPSL.

## 2. Patients and Methods

A retrospective analysis of dermoscopic images of histopathologically diagnosed clinically challenging flat FPSLs was conducted.

### 2.1. Patients

Sixty-two patients (23 males and 39 females; mean age 55.2; age range 25–70 years) with newly developed 64 untreated FPSLs were included in this study, after obtaining an informed written consent. The study was conducted according to the Declaration of Helsinki Principles and was approved by the medical ethical committee of Donetsk Medical National University, Ukraine. Patients were collected from dermatologic departments of Donetsk Medical National University, Ukraine. They were evaluated according to clinical examination, dermoscopic examination, and histopathological examination in dermatologic departments of Donetsk Medical National University, Ukraine, and Queen Medical, Qatar. According to histopathological diagnosis, lesions were further divided into 4 groups: group I: 24 SKs, group II: 17 AKs, group III: 8 LM, and group IV: 15 SLs.

Patients, who started treatment or were on any kind of topical applications likely to alter the results of the study (e.g., topical retinoids), were excluded from the study.

### 2.2. Methods

#### 2.2.1. Clinical Evaluation

All patients were subjected to clinical evaluation including history with attention to smoking and occupations and/or hobbies with excessive sun exposure, duration of the lesions, relation to sun exposure, previous therapies, history of relevant medical conditions, for example, photosensitivity, relevant surgical history including dermabrasion and laser surgery, and previous or present relevant medication such as retinoids. Complete general and dermatological examinations were done. Lesions were clinically evaluated regarding site, size, color, shape, and surface. They varied in size between patients (from 6 mm up to 28 mm) and varied in color from light to dark brown.

Photographic documentation was done using identical camera setting and lighting by a Canon EOS 450D digital camera.

#### 2.2.2. Histopathological Diagnosis

Four millimeter punch skin biopsies were collected from skin lesions of all patients. Biopsy specimens were submerged immediately in 10% buffered formalin, prepared for paraffin embedding and paraffin blocks, and sectioned in 4-micron thick sections mounted on glass slides and prepared for routine hematoxylin and eosin (H&E) stain for conventional histopathology, diagnosis, and categorizing the patients into the above-mentioned groups. All slides were coded before analysis and read blindly to ensure competent evaluation of the findings.

#### 2.2.3. Dermatoscopy

Patients underwent dermoscopy (Heine Delta K-256.10.118, Heine Optotechnik, Germany) and photography prior to biopsy taking. We assessed the photographs retrospectively to establish the dermoscopic findings corresponding to the four aforementioned histopathologically proven diagnoses.

Four main dermoscopic features were evaluated, namely, sharp demarcation, pigment pattern (including network, dots/globules, pseudonetwork, negative network, and homogenous structureless pigmentation), follicular/epidermal pattern (including milia-like cysts, cerebriform pattern, and comedo-like openings), and vascular patterns (including coma shaped vessels, hairpin vessels, arborizing vessels, crown vessels, linear vessels, branched vessels, lacunae, glomerular vessels, and dotted vessels).

#### 2.2.4. Statistical Analysis

Data were statistically described in terms of range: mean ± standard deviation (SD), median, frequencies (number of cases), and relative frequencies (percentages) when appropriate. Taking into account the small number of cases in subgroups (less than 30), statistical calculation was performed using Pearson's chi-squared test. According to Pearson's chi-squared test statistically significant difference in distribution of feature groups was considered if *P* < 0.05. Pairwise comparisons of the relative frequency of occurrence of dermoscopic features in the groups (%) were performed using Fisher's exact test. All statistical calculations were done using computer programs, Microsoft Excel version 7 (Microsoft Corporation, NY, USA), and SPSS (Statistical Package for the Social Science; SPSS Inc., Chicago, IL, USA) version 15 for Microsoft Windows.

## 3. Results and Discussion

Table 1 demonstrates the frequency of detected dermoscopic features in the studied groups of lesions.

**Figure 1:**
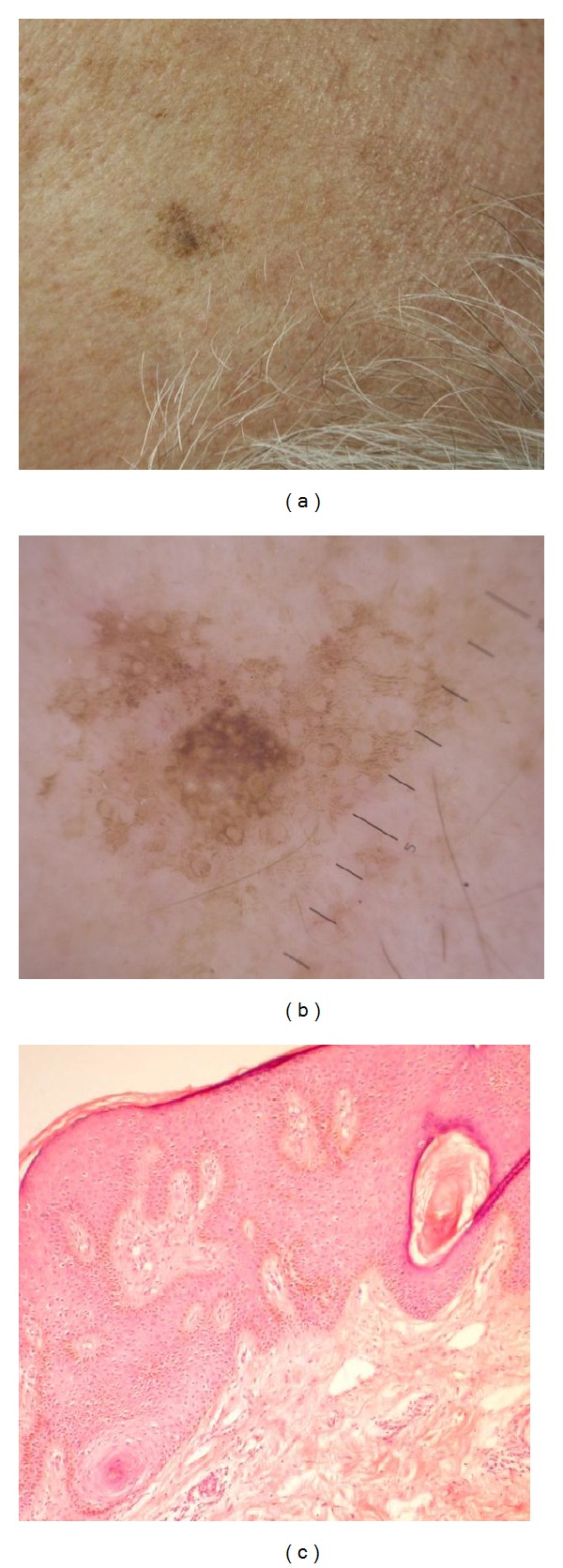
(a) Seborrheic keratosis, temporal area (male, 67 years). (b) Same lesion dermoscopy: fissuring in the form of fingerprint-like structures around follicles, milia-like cysts, and comedo-like opening in the center of lesion. (c) Histopathology of seborrhoeic keratosis (acanthotic type) showing irregular epidermal hyperplasia mainly in the form of acanthosis (H&E ×200).

In group I (SK), the most specific dermoscopic features are follicular/epidermal pattern (cerebriform pattern in 100% of lesions, milia-like cysts in 50%, and comedo-like openings in 37.50%) and sharp demarcation (in 54.17% of lesions). Thus, the main dermoscopic feature was different types of fissures which can be described as ridges, fingerprint-like structures, “fat fingers,” or cerebriform pattern. Despite the large range of descriptive words, all of them correspond to the same histological features of epidermal acanthosis and different degree of melanization of keratinocytes (Figure 1).

**Figure 2:**
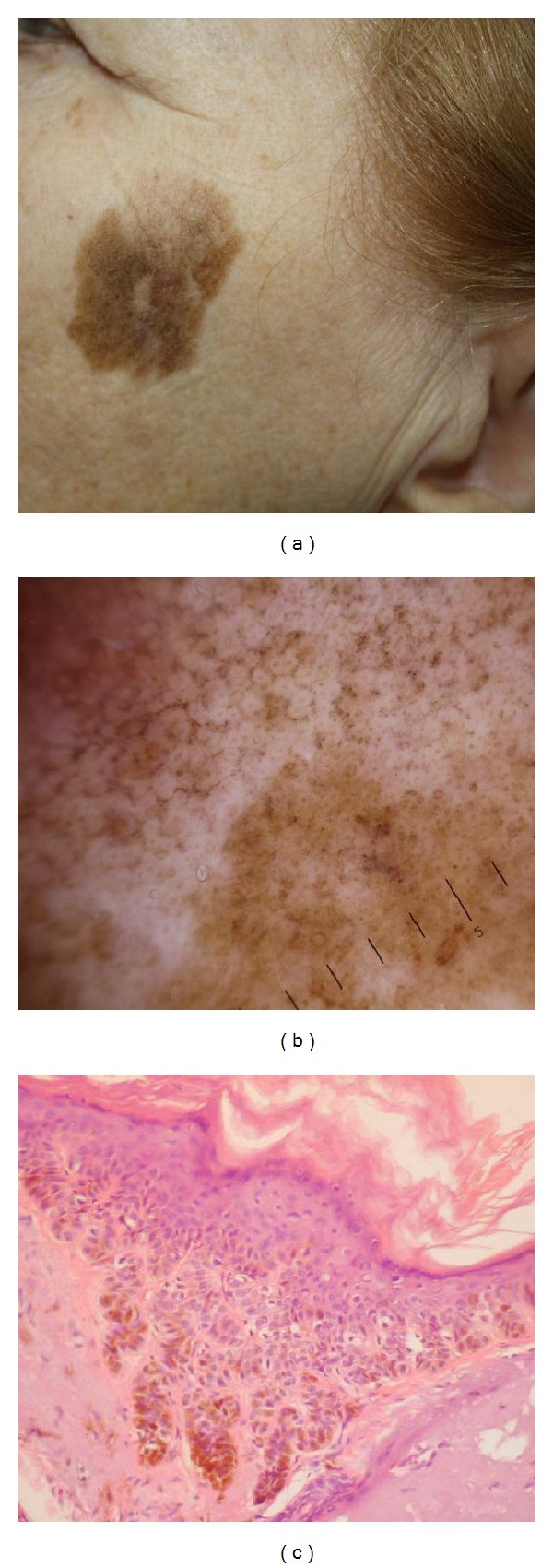
(a) Actinic keratosis, cheek zone (female, 58 years). (b) Same lesion dermoscopy: pseudonetwork, brown dots and globules, homogenous structureless pigmentation. (c) Actinic keratosis histopathology with epidermal acanthosis and parakeratosis. Keratinocytes show increased melanization and some disorganization with enlarged hyperchromatic pleomorphic nuclei. Solar elastosis is evident in the papillary dermis (H&E ×200).

AK lesions (group II) showed prominent pigment pattern (pseudonetwork in 52.94% of lesions, dots and globules in 47.06%, and homogenous structureless pigmentation in 47.06%) and vascular pattern, mainly in the form of perifollicular/crown pattern (in 41.18%). These findings correspond to increased irregular melanization of keratinocytes in histopathology (Figure 2). Vascular pattern was detected mainly in the form of perifollicular/crown pattern (in 41.18%).

**Figure 3:**
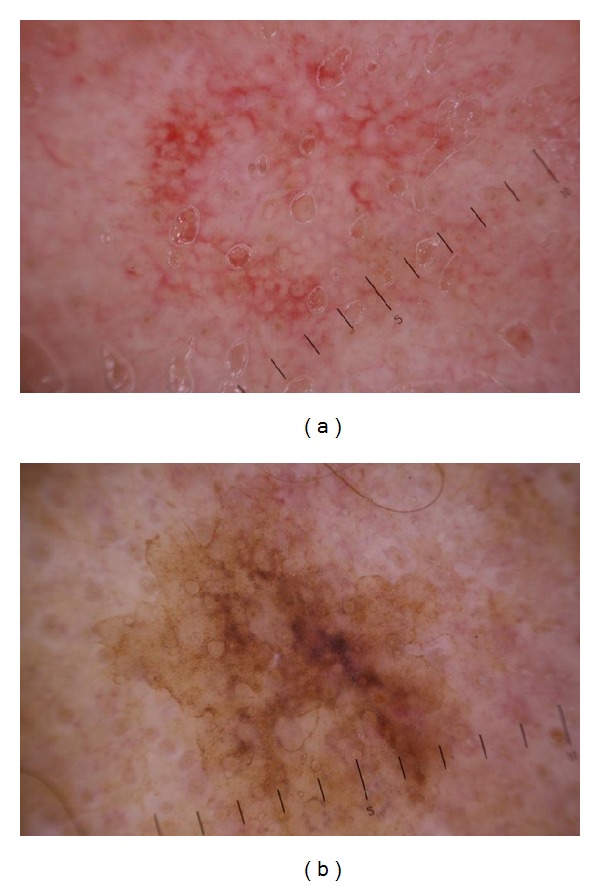
Dermoscopy of actinic keratosis versus lentigo maligna. (a) Actinic keratosis, vascular pattern in the form of perifollicular crown (strawberry pattern) mixed with slightly visible pigment pseudonetwork. (b) Lentigo maligna dermoscopy; “strawberry pattern” is covered by pseudonetwork and homogenous structureless pigmentation.

Despite having the most dermoscopically polymorphous lesions comparing with SK and SL, AK (group II) and LM (group IV) showed a composite characteristic pattern named “strawberry pattern” in 41.18% and 25% of lesions, respectively, characterized by a background erythema and red pseudonetwork consisting of unfocused, large vessels located between the hair follicles, associated with prominent follicular openings surrounded by a white halo. The difference between “strawberry pattern” in AK and LM is that in AK “strawberry pattern” is more constant and/or mixed with pseudonetwork (52.94%) (Figure 3(a)), while in LM “strawberry pattern” is widely covered by pseudonetwork (87.5%), homogenous structureless pigmentation (75%), and other vascular patterns (coma shaped, arborizing vessels, etc.) (Figure 3(b)).

**Figure 4:**
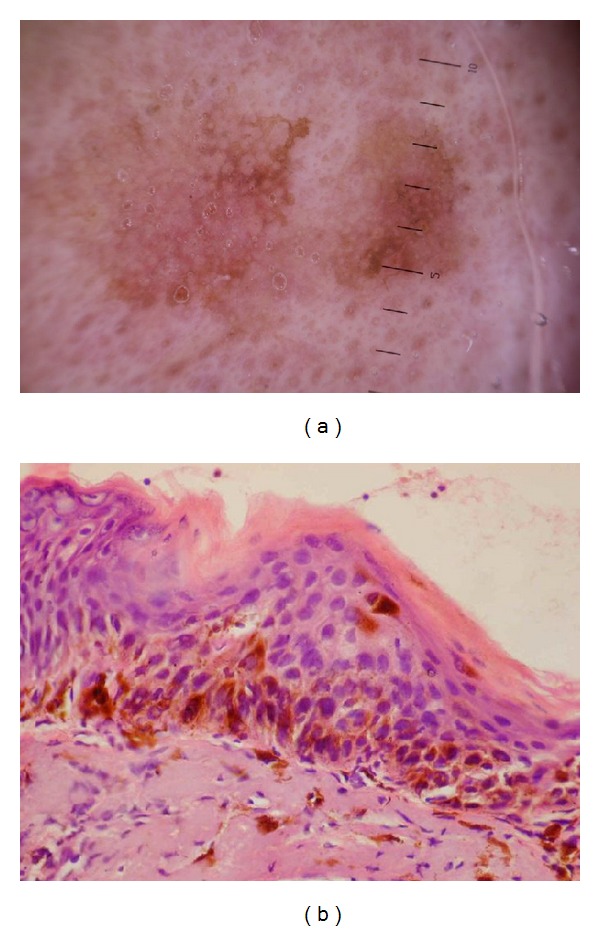
(a) Lentigo maligna dermoscopy; irregular pigment pseudonetwork covers vascular structures. Homogenous structureless pigmentation is present. (b) Lentigo maligna histopathology showing proliferation of atypical melanocytes with different shape and size in epidermis and dermoepidermal junction (H&E ×400).

Overall, LM lesions had more than 3 dermoscopic features—vascular and/or pigment components. Regarding the pigment components, we detected 3 of the 4 classic criteria developed by Schiffner et al. [3] for early recognition of LM, namely, pseudonetwork and pigmented dots and globules. Pralong et al. [4] described three additional original vascular criteria at a relatively high frequency in LM: increased density of the vascular network (58%), red rhomboidal structures (40%), and target-like patterns (41%). Despite being detected in 62.5% of our lesions, we found that vascular component of LM did not show certain specific characteristic features, as it showed one or more of the following patterns in each lesion: coma shaped vessels, arborizing vessels, linear vessels, lacunae, hairpin vessels, branched vessels, and crown vessels. Further studies on larger population of patients are needed to establish diagnostic vascular dermoscopic criteria. Histopathologically, LM showed isolated proliferation of atypical melanocytes with different shape and size in epidermis and dermoepidermal junction (Figure 4).

**Figure 5:**
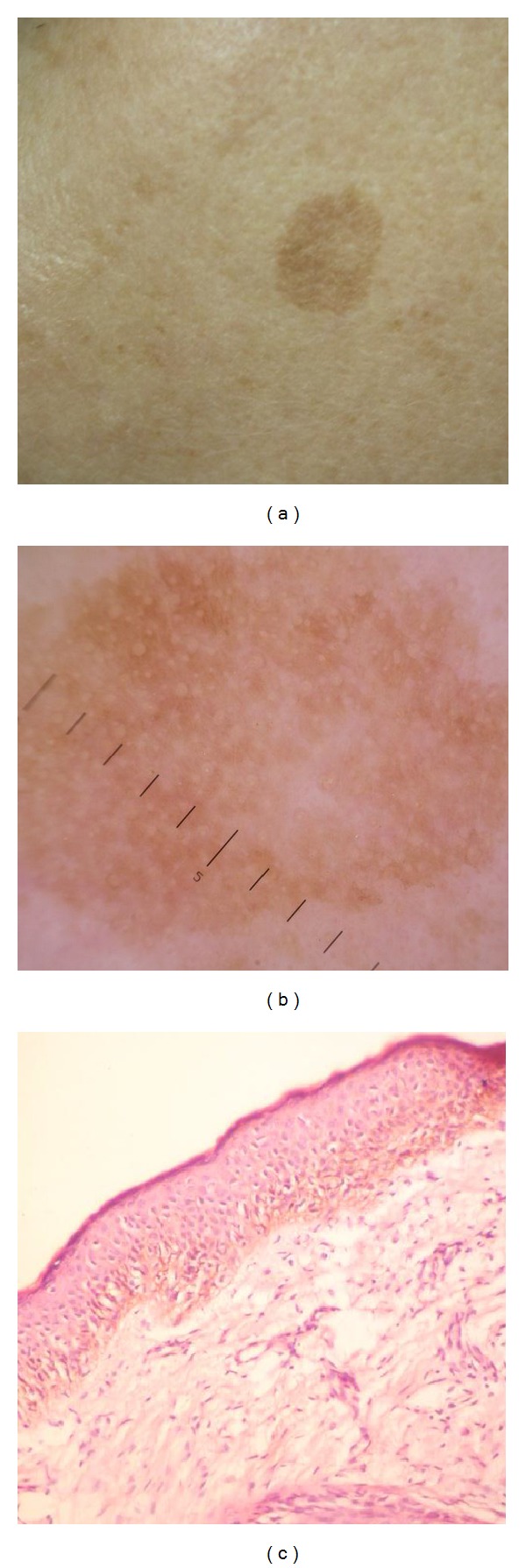
(a) Solar lentigo on the cheek (female, 52 years). (b) Same lesion dermoscopy: structureless homogenous pigmentation and numerous openings of hair follicle ostia and adnexal structures. (c) Solar lentigo histopathology showing hyperkeratosis, slight keratinocytes disorganization, and melanization (H&E ×200).

In group III (SL), structureless homogenous pigmentation was recognized in all lesions (100%), corresponding to regular pigment charging of keratinocytes (Figure 5). Only 3 (20.0%) SL in our study had sharp demarcation, despite being detected easily in all lesions on clinical examination.

According to obtained data, 6 from the studied 16 features showed statistically significant different expression in the studied groups of lesions, namely, sharp demarcation, pigment dots and globules, pseudonetwork, homogenous structureless pigmentation, milia-like cysts, and cerebriform structures (*P* < 0.05 using Pearson's chi-squared test) (Table 2).

Observed in 54.17% of SK cases, 20% of SL cases, and 17.65% of AK cases, sharp demarcation was the most constant and statistically significant dermoscopic feature in the group of SK compared to SL and AK using Fisher's exact test (φ*) (*P* = 0.005 and *P* = 0.013, resp.). Pigment dots and globules have been observed in 50% of LM cases, 47.06% of AK cases, 16.67% of SK cases, and 13.33% of SL cases, with no statistically significant difference between AK and LM (they cannot be considered as prominent differentiating feature between AK and LM). Moreover, the presence of this pattern in SK and SL was too weak to consider it diagnostic, despite the presence of statistically significant difference between AK and SK; AK and SL (*P* = 0.017 and *P* = 0.015), as well as between LM and SK; LM and SL (*P* = 0.037 and *P* = 0.03). Pseudonetwork was more or less present in all of studied dermatoses: 87.5% of LM cases, 52.94% of AK cases, 40% of SL cases, and 4.17% of SK cases. Comparing all of them, there were statistically significant differences apart from comparing AK with SL. Homogenous structureless pigmentation was observed in 100% of SL cases, 75% of LM cases, 47.06% AK cases, and 8.33% of SK cases, and statistical analysis denoted that this dermoscopic feature is statistically significant in all studied dermatoses (*P* < 0.05). However it is more constant feature in SL and LM (*P* = 0.007) compared to SK (*P* < 0.001). Comparing the frequency of millia-like cysts in SK with LM, they were detected in 50% of SK cases and 12.5% of LM cases, with a statistically significant difference (*P* < 0.019). Therefore, the presence of milia-like cysts can be considered as a constant dermoscopic feature of SK. However, the most constant dermoscopic pattern for SK was cerebriform structures which were observed in 100% of SK cases, despite that few of them were also observed in LM (12.5%) (*P* < 0.001) (Table 2).

**Figure 6:**
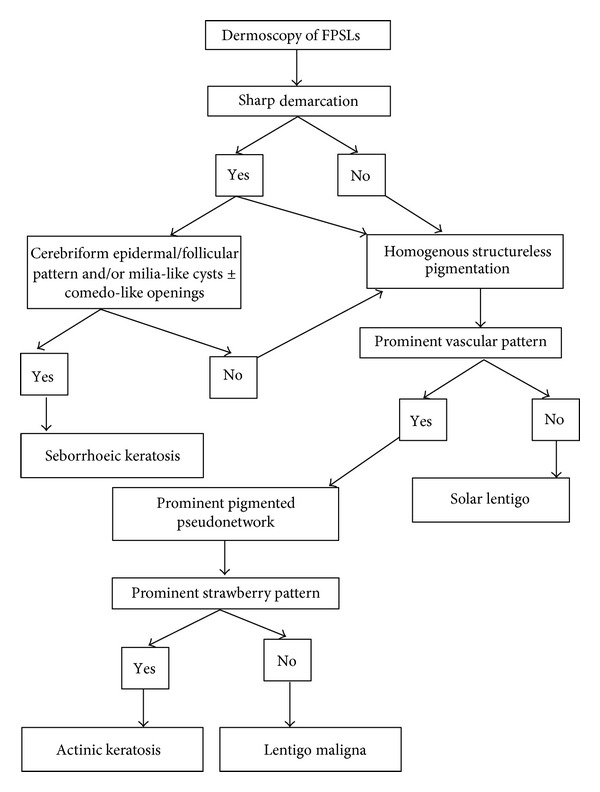
Algorithm for diagnostic dermoscopic features of FPSLs.

**Figure 7:**
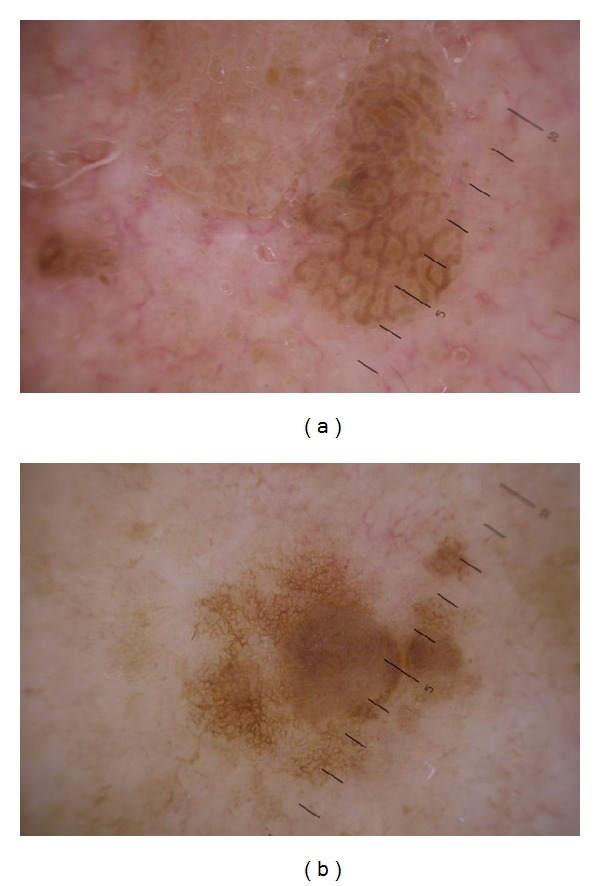
Seborrheic keratosis dermoscopy: (a) cerebriform pattern (brain-like appearance) and (b) fingerprint-like and cerebriform structures in the same lesion.

From the above-mentioned data, we developed an algorithm to be used as guide in diagnostic features of FPSLs (Figure 6). We found that SK is characterized by sharp demarcation. In addition, follicular/epidermal pattern is the main feature among other dermoscopic features, which is due to the nature of SK as an epidermal lesion. Despite the fact that sometimes we can see milia-like cysts (corresponding to intraepidermal keratin-filled cysts in histopathology) and comedo-like openings (corresponding histopathologically to keratin-filled invaginations of the epidermis) in BCC, melanocytic nevi, and even AK, fissuring (irregular linear keratin-filled depressions) which can lead to cerebriform pattern (brain-like appearance) in hypertrophic SK or “fat fingers” in flat SK is the most prominent dermoscopic feature which can serve the principal diagnostic feature (Figure 7) (see algorithm in Figure 6).

In early stage of AK vascular pattern is prominent. It is presented as numerous perifollicular crowns. The good developed reticulation is shown as “strawberry pattern” (vascular pseudonetwork) (Figure 3(a)). Strawberry pattern can be mixed with pigment pattern (pseudonetwork), which results from the unique anatomy of the facial skin that is devoid of rete ridges and contains abundant, closely located follicular infundibula [5–7], dots and globules, and homogenous structureless pigmentation. Zalaudek et al. (2012) [8] stated that ared pseudonetwork was significantly associated with AK. However, existence of pseudonetwork can be very confusing with LM (Figure 4). Akay et al. (2010) [9] showed that pigmented AK has a striking similarity to LM in clinical and dermatoscopic features, thus representing a diagnostic challenge. Eleven essential dermatoscopic features were observed in their patients' facial AK: slate-grey dots (70%), annular-granular pattern (39%), rhomboidal structures (36%), pseudonetwork (36%), black globules (34%), slate-grey globules (33%), black dots (30%), asymmetrical pigmented follicular openings (25%), hyperpigmented rim of follicular openings (21%), slate-grey areas (18%), and streaks (3%). All dermatoscopic findings except black blotches were observed in pigmented AK. According to Pock et al. (2007) [10], the pigmented atypical melanocytes' role in LM presenting as black dots in the dermatoscopical picture was displayed by the individually pigmented keratinocytes in pigmented AK. The groups of melanophages presenting as gray dust were present in pigmented AK similarly to their presentation in LM. The character of the pigmented pseudo-network may be the same in both afflictions. However, an atypical pseudonetwork, characterized by wide, black to brown meshes and irregular holes known to represent the uneven descent of neoplastic melanocytic cells into individual hair follicles [1, 3, 6, 11], is indicative of LM [12]. In addition, slate gray dots and globules detected in pigmented AK have a more uniform size and a more regular distribution within the lesion than do those found in LM. Finally, the presence of hypopigmented follicular openings surrounded by a rim of hyperpigmentation is more frequent in LM but not in AK [3, 6].

Dermoscopic differential diagnosis between pigmented SK and pigmented AK on the face is not usually easy. In contrast to AK, SK on the face is distinguished by a typical pigmented pseudonetwork with regular meshes and holes, opaque areas, pseudofollicular openings, and horny pseudocysts [6, 7, 13]. Overlapping SK and AK is not so often, and the fingerprint pattern is usually the main feature to recognize SK.

SL is presented as an intensification of pigment pattern which forms mostly simple homogenous structureless feature or pseudonetwork but never prominent vascular pattern (see algorithm in Figure 6) (Figure 5). In SL the fingerprint pattern is not the constant feature, and mostly we can observe small interrupted focus of “fingerprint” on the homogenous structureless field [2]. In some cases, solar lentigo (*syn*. lentigo senilis) on the face may clinically simulate pigmented AK. Dermoscopic features of solar lentigo include delicate light brown typical pseudonetwork and a regular diffuse pigmentation.

## 4. Conclusion

Taken all together, we developed an algorithm to be used as a guide in diagnostic features of FPSLs. Sharp demarcation with cribriform pattern is characteristic of SK, while homogenous structureless pigmentation without prominent vascular pattern is characteristic of SL. AK is characterized by prominent strawberry pattern. On the other hand, homogenous structureless pigmentation and vascular pattern with pseudonetwork are seen in LM, lacking of prominent strawberry pattern.

## Figures and Tables

**Table 1:** The frequency of the studied dermoscopic features in different lesions.

	Seborrhoeic keratosis Number: 24	Actinic keratosis Number: 17	Solar lentigo Number: 15	Lentigo maligna Number: 8
Sharp demarcation	**13 (54.17%)**	3 (17.65%)	3 (20.00%)	**0 (0%)**
Pigment patterns				
(i) Network	1 (4.17%)	1 (5.88%)	2 (13.33%)	0 (0%)
(ii) Dots/globules	4 (16.67%)	**8 (47.06%)**	2 (13.33%)	4 (50.0%)
(iii) Pseudonetwork	1 (4.17%)	**9 (52.94%)**	6 (40.00%)	**7 (87.50%)**
(iv) Negative network	0 (0%)	0 (0%)	0 (0%)	0 (0%)
(v) Homogenous structureless	2 (8.33%)	**8 (47.06%)**	**15 (100%)**	**6 (75.00%)**
Follicular/epidermal patterns				
(i) Milia-like cysts	**12 (50%)**	0 (0%)	0 (0%)	1 (12.5%)
(ii) Cerebriform pattern	**24 (100.0%)**	0 (0%)	0 (0%)	1 (12.5%)
(iii) Comedo-like openings	**9 (37.5%)**	3 (17.65%)	0 (0%)	1 (12.5%)
Vascular patterns				
(i) Coma shaped	3 (12.5%)	3 (17.65%)	0 (0%)	3 (37.5%)
(ii) Hairpin	7 (29.17%)	2 (11.76%)	0 (0%)	0 (0%)
(iii) Arborizing	1 (4.17%)	1 (5.88%)	3 (20.0%)	2 (25.0%)
(iv) Perifollicular (crown)	0 (0%)	**7 (41.18%)**	0 (0%)	2 (25.0%)
(v) Linear	2 (8.33%)	2 (11.76%)	1 (6.67%)	1 (12.5%)
(vi) Branched	4 (16.67%)	4 (23.53%)	1 (6.67%)	1 (12.5%)
(vii) Lacunae (red lagoons)	0 (0%)	2 (11.76%)	0 (0%)	1 (12.5%)
(viii) Glomerular	0 (0%)	0 (0%)	0 (0%)	0 (0%)
(ix) Globular (dotted)	0 (0%)	3 (17.65%)	0 (0%)	0 (0%)

**Table 2:** The relative frequency of occurrence of dermoscopic features using Fisher's exact test.

Dermoscopic features	Groups				The statistical significance of differences (*P*) between groups					
	AK	SK	SL	LM	AK-SK	AK-SL	AK-LM	SK-SL	SK-LM	SL-LM
Sharp demarcation	17.65	**54.17**	20	0	**0.005**	>0.05	—	**0.013**	—	—
Dots and globules	47.06	16.67	13.33	50	**0.017**	0.015	>0.05	**>0.05**	0.037	0.03
Pseudonetwork	52.94	4.17	40	87.5	**<0.001**	>0.05	0.033	**<0.001**	<0.001	0.007
Homogenous structureless pigmentation	47.06	8.33	**100**	**75**	**<0.001**	**<0.001**	>0.05	**<0.001**	**<0.001**	**0.007**
Milia-like cysts	0	**50**	0	12.5	>0.05	—	>0.05	—	**0.019**	—
Fissure	0	**100**	0	12.5	>0.05	—	>0.05	—	**<0.001**	—

## References

[1] Stante M, Giorgi V, Stanganelli I, Alfaioli B, Carli P (2005). Dermoscopy for early detection of facial lentigo maligna. *British Journal of Dermatology*.

[2] Malvehy J, Puig S, Braun RP (2006). *Handbook of Dermoscopy*.

[3] Schiffner R, Schiffner-Rohe J, Vogt T (2000). Improvement of early recognition of lentigo maligna using dermatoscopy. *Journal of the American Academy of Dermatology*.

[4] Pralong P, Bathelier E, Dalle S (2012). Dermoscopy of lentigo maligna melanoma: report of 125 cases. *British Journal of Dermatology*.

[5] Argenziano G, Soyer HP, Chimenti S (2003). Dermoscopy of pigmented skin lesions: results of a consensus meeting via Internet. *Journal of the American Academy of Dermatology*.

[6] Stolz W, Schiffner R, Burgdorf WHC (2002). Dermatoscopy for facial pigmented skin lesions. *Clinics in Dermatology*.

[7] Stolz W, Braun-Falco O, Bilek P (2002). *Color Atlas of Dermatoscopy*.

[8] Zalaudek I, Giacomel J, Schmid K (2012). Dermatoscopy of facial actinic keratosis, intraepidermal carcinoma, and invasive squamous cell carcinoma: a progression model. *Journal of the American Academy of Dermatology*.

[9] Akay BN, Kocyigit P, Heper AO, Erdem C (2010). Dermatoscopy of flat pigmented facial lesions: diagnostic challenge between pigmented actinic keratosis and lentigo maligna. *British Journal of Dermatology*.

[10] Pock L, Drlík L, Hercogová J (2007). Dermatoscopy of pigmented actinic keratosis—a striking similarity to lentigo maligna. *International Journal of Dermatology*.

[11] Cognetta AB, Stolz W, Katz B, Tullos J, Gossain S (2001). Dermatoscopy of lentigo maligna. *Dermatologic Clinics*.

[12] Schiffner R, Schiffner-Rohe J, Vogt T (2000). Improvement of early recognition of lentigo maligna using dermatoscopy. *Journal of the American Academy of Dermatology*.

[13] Stante M, Giorgi V, Stanganelli I, Alfaioli B, Carli P (2005). Dermoscopy for early detection of facial lentigo maligna. *British Journal of Dermatology*.

